# A retrospective analysis of hyponatremia during terlipressin treatment in patients with esophageal or gastric variceal bleeding due to portal hypertension

**DOI:** 10.1002/jgh3.12254

**Published:** 2019-10-21

**Authors:** Xv Han, Jia Li, Ji‐Ming Yang, Min Gao, Lei Wang

**Affiliations:** ^1^ Tianjin Second People's Hospital Tianjin Hepatopathy Research Institute Tianjin China

**Keywords:** endogenous preconditioning, hyponatremia, terlipressin

## Abstract

**Background and Aim:**

To explore the risk factors of hyponatremia caused by terlipressin.

**Methods:**

Forty‐four patients with acute variceal bleeding treated with terlipressin from December 2016 to December 2018 were analyzed.

**Results:**

During the treatment, serum sodium levels decreased from 137.78 to 126.59 mmol/L (*P* < 0.05), with an average decrease of 11.19 mmol/L. The serum sodium level decreased by less than 5 mmol/L in 12 patients (27.27%), by 5–10 mmol/L in 13 patients (27.27%), and by more than 10 mmol/L in 19 patients (43.18%). The difference in baseline serum sodium levels was statistically significant (*P* < 0.05), and the differences in baseline total bilirubin levels, Child‐Pugh scores, and model for end‐stage liver disease scores were also significant. Logistic regression analysis suggested that the initial sodium level was an independent risk factor for the decrease in the serum sodium concentration caused by terlipressin.

**Conclusion:**

The incidence of hyponatremia is not low during treatment with terlipressin; a higher baseline serum sodium level is a risk factor for hyponatremia during treatment with terlipressin, and the mechanism may be related to endogenous vasopressin preconditioning.

## Graphical abstract

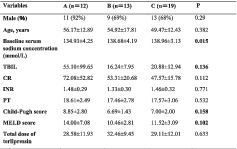

A retrospective analysis was conducted of hyponatremia during terlipressin treatment in patients with esophageal or gastric variceal bleeding due to portal hypertension. Forty‐four patients with acute variceal bleeding treated with terlipressin from December 2016 to December 2018 were analyzed.

## Introduction

Esophagogastric variceal bleeding (EVB) is one of the most dangerous complications in patients with portal hypertension due to liver cirrhosis. Terlipressin has been listed as the first‐line treatment for EVB as it can shrink visceral vessels and reduce portal pressure.^1^, ^2^, ^3^ In recent years, severe hyponatremia has been observed in some patients during treatment with terlipressin. Thirty‐six patients with acute variceal bleeding treated with terlipressin were analyzed to explore the risk factors for hyponatremia caused by terlipressin and the underlying mechanism.

## Methods

We retrospectively reviewed the medical records of 44 inpatients who had been treated with terlipressin for variceal bleeding from December 2016 to December 2018. Endoscopic therapy was used in most patients at the time of diagnosis. All patients were treated with terlipressin before endoscopic therapy. In addition to terlipressin, the patients were treated according to the current standard methods. All enrolled patients initially received 2 mg of terlipressin at 4‐h intervals. Terlipressin treatment was reduced to 1 mg/4 h after 24 h of no bleeding and was maintained at 1 mg/12 h after no active bleeding, with a course of 3–7 days. Blood pressure, heart rate, electrolytes, and liver and kidney function were monitored every 2 days.

Severe hyponatremia (<120 mmol/L) or marked reductions in serum sodium levels with the development of neurological manifestations indicate the need for a reduction in the dose of terlipressin or withdrawal and replacement with stilamin. If the serum sodium level continues to decrease, hypertonic saline can be administered, but the rate of increase in the serum concentration of sodium should be moderated to avoid osmotic demyelination syndrome.^4^ To evaluate the effect of treatment on the serum sodium concentration, the patients were divided into three groups according to the difference between the baseline and lowest serum concentrations of sodium during the 7 days of therapy, as follows: group A, either no change or an increase or decrease of less than 5 mmol/L; group B, a decrease between 5 and 10 mmol/L; and group C, a decrease of greater than 10 mmol/L.

### *Statistical analysis*

Complete follow‐up data were available for all patients. Statistical analysis was performed using SPSS, version 23 (IBM Corp.). Continuous variables are presented as the mean ± SD values, and categorical variables are presented as frequencies (%). Univariate analyses of the variables associated with the development of a reduction in the serum sodium concentration were carried out by means of one‐way analysis of variance (for continuous variables). A multivariate logistic regression model was used to select the best predictor of hyponatremia. A *P* value less than 0.05 was considered statistically significant.

## Results

A total of 44 patients were enrolled in this study. The mean age was 52.91 years, and 75% of the patients were male. During treatment, the serum sodium level decreased from 137.78 to 126.59 mmol/L (*P* < 0.05), with an average decrease of 11.19 mmol/L. During treatment, the serum sodium levels of 12 patients (27.27%) decreased by less than 5 mmol/L; these patients constituted group A. The serum sodium levels of 13 patients (29.55%) decreased by 5–10 mmol/L; these patients were in group B. The serum sodium levels of 19 patients (43.18%) decreased by more than 10 mmol/L, and these patients comprised group C. When one patient's serum sodium concentration decreased from 135 to 117 mmol/L early in the treatment course (<48 h), and neurological manifestations, such as restlessness and the inability to cooperate with the infusion, were observed, a sedative was administered, after which the patient was able to cooperate with the infusion. Terlipressin was withdrawn and replaced by somatostatin to reduce portal pressure; as the serum sodium continued to decrease, we administered hypertonic saline. After 2 days, the patient's serum sodium level gradually returned to normal, and he regained consciousness.

The indices were compared in the different groups stratified by the amount by which the serum sodium level decreased (Table 1).

**Table 1:** The indices were compared in the different groups (A,B,C)

Variables	A (*n* = 12)	B (*n* = 13)	C (*n* = 19)	*P*
Male (%)	11 (92)	9 (69)	13 (68)	0.29
Age (years)	56.17 ± 12.89	54.92 ± 17.81	49.47 ± 12.43	0.382
Baseline serum sodium concentration (mmol/L)	134.93 ± 4.25	138.68 ± 4.19	138.96 ± 3.13	0.015
TBIL	55.10 ± 99.65	16.24 ± 7.95	20.88 ± 12.94	0.136
CR	72.08 ± 52.82	53.31 ± 20.68	47.57 ± 15.78	0.112
INR	1.48 ± 0.29	1.33 ± 0.30	1.46 ± 0.32	0.771
PT	18.61 ± 2.49	17.46 ± 2.78	17.57 ± 3.06	0.532
Child‐Pugh score	8.85 ± 2.80	6.69 ± 1.43	7.00 ± 2.00	0.158
MELD score	14.00 ± 7.08	10.46 ± 2.81	11.52 ± 3.09	0.102
Total dose of terlipressin	28.58 ± 11.93	32.46 ± 9.45	29.11 ± 12.01	0.633

△Na < 5 mmol/L (group A), 5 mmol/L ≤ △Na≤10 mmol/L (group B) and △Na > 10 mmol/L (group C). Differences in baseline serum sodium levels among the three groups were statistically significant (*P* < 0.05), as were the differences in TBIL levels, Child‐Pugh scores, and MELD scores.

CR, creatinine; INR, international normalized ratio; MELD, model for end‐stage liver disease; PT, prothrombin time; TBIL, total bilirubin.

Logistic regression analysis of risk factors was performed for serum sodium reduction (Table 2).

**Table 2:** Logistic regression analysis of risk factors for serum sodium reduction

Items	OR	95% CI		*P*
Na0	0.74	0.57	0.98	0.02
Child‐Pugh score	1.12	0.58	2.49	0.28
MELD score	0.80	0.46	1.27	0.26
TBIL	1.04	0.95	1.13	0.13

CI, confidence interval; MELD, model for end‐stage liver disease; TBIL, total bilirubin; OR, odds ratio.

## Discussion

Terlipressin is a synthetic vasopressin analogue. Vasopressin, also known as antidiuretic hormone (ADH), has vasoconstrictive and antidiuretic effects. Three glycidyl ligands at the N‐terminal of terlipressin are excised by the amino peptide enzyme in the body at a stable rate, and active lysine vasopressin is slowly released.^5^

The mechanism by which terlipressin leads to hyponatremia may be as follows: by binding to the V1 receptor on vascular smooth muscle, terlipressin can contract blood vessels (mainly visceral blood vessels), leading to the redistribution of blood flow and contributing to the increase in arterial blood pressure and renal perfusion^6^ and the increase of sodium hydrochloride excretion in patients.^7^ In the rat model of cirrhotic ascites, terlipressin can increase arterial blood pressure, reduce ascites, and increase the excretion rate of sodium urine. This phenomenon is basically similar to the effect of a V1 receptor stimulator, suggesting that the mechanism of terlipressin constricting blood vessels mainly exerts biological effects by activating the V1 receptor on the visceral blood vessels.^8^ In addition to its activity on the V1 receptors, terlipressin also activates the V2 receptors of the basal lateral membrane of the main cells of the renal collecting duct, elevates the levels of cyclic adenosine monophosphate (cAMP), stimulates protein kinase A, activates AQP 2, opens water channels, and allows the collection tube to absorb a large amount of free water. Water permeability increases by 8–10 times, which could lead to dilution hyponatremia.^9^ Nevertheless, the potential antidiuretic effects of terlipressin therapy have received little or no attention in clinical practice.

The results of this study showed that 16 patients (44.4%) developed severe hyponatremia during treatment with terlipressin. Moreover, the difference in serum sodium concentrations before and after terlipressin administration was statistically significant. The results of this study were similar to those of the studies by Yim *et al*.^10^ and Solà *et al*.,^11^ in which the incidence of severe hyponatremia was 36–38.8%. Different incidences of hyponatremia may be associated with different baseline conditions in the enrolled patients. The incidence of hyponatremia is not low during treatment with terlipressin.

The results of this study suggested that the differences in baseline serum sodium levels were statistically significant (*P* < 0.05), and the baseline total bilirubin (TBIL) levels, Child‐Pugh scores, and model for end‐stage liver disease (MELD) scores were also different. Logistic regression analysis indicated that the initial sodium level was an independent risk factor for the decrease in serum sodium concentration caused by terlipressin, and the higher the initial sodium level, the more likely hyponatremia was to appear. Yim *et al*.^10^ believed that hyponatremia often occurs when terlipressin is applied, and the occurrence of hyponatremia was related to young age, low Child‐Pugh score, low body weight, high initial sodium level, and prolonged use of terlipressin. However, our study showed no significant difference in age and total terlipressin use between the different groups. In our study, due to the small number of enrolled cases, there may be statistical errors in the logistic regression analysis. Solà *et al*.^11^ also conducted a retrospective analysis, finding that only the MELD score was an independent risk factor for death, and the initial level of serum sodium and the degree of serum sodium decline were independent of the prognosis. In the study by Solà *et al*.,^11^ many patients were treated with terlipressin because somatostatin treatment was ineffective, and the included patients were screened imperceptibly, which may have affected the results of the study. Kang *et al*.^12^ retrospectively analyzed the incidence rate and risk factors of decreased serum sodium levels in 127 patients with liver cirrhosis after the application of terlipressin. Univariate analysis showed that age, serum creatinine level, MELD score, and other factors were significantly correlated with the decrease in the serum sodium concentration, but multivariate analysis showed that only the initial sodium level was a powerful predictor of the reduction in serum sodium induced by terlipressin, which was consistent with the findings of our study.

The above studies suggested that higher baseline serum sodium levels and better liver function (low MELD and Child‐Pugh scores) were risk factors for hyponatremia during treatment with terlipressin, although the mechanism remains unclear. As a result of decompensated liver cirrhosis portal vein pressure, vasopressin release may be increased, leading to the speculation that, in patients with decompensated cirrhosis of the liver, endogenous vasopressin preconditioning may be present due to the increase in endogenous vasopressin occupying the V2 receptor or renal collecting duct and the decrease in AQP‐2 expression, weakening the antidiuretic effect.^10^ In addition, it is also believed that the increase in urinary sodium caused by the activation of the V1 receptor by terlipressin is also a cause of hyponatremia.^13^, ^14^ The mechanism remains to be elucidated.

During the treatment of portal hypertensive hemorrhage in cirrhosis with terlipressin, a sharp decrease in serum sodium concentration is common, especially in patients with better basic liver function and higher baseline sodium levels. Some patients are severely ill with neurological complications, which are usually reversible after the withdrawal of terlipressin. Therefore, the serum sodium level should be closely monitored during the treatment of portal hypertensive hemorrhage of cirrhosis with terlipressin, and the occurrence of hyponatremia should be promptly detected and corrected.

This study was limited by its retrospective nature and a small number of patients; hence, the findings highlighted here need further validation with a multicenter prospective study.
